# D‐Carvone Mitigating Renal Damage and Enhancing Antioxidant Defense in Lithium Induced Nephrotoxicity

**DOI:** 10.1002/fsn3.70830

**Published:** 2025-09-26

**Authors:** Semin Gedikli, Deniz Tekiner, Hülya Kara, Saime Özbek Şebin, Tubanur Aslan Engin

**Affiliations:** ^1^ Department of Histology and Embryology, Faculty of Veterinary Atatürk University Erzurum Türkiye; ^2^ Department of Anatomy, Faculty of Veterinary Atatürk University Erzurum Türkiye; ^3^ Department of Physiology, Faculty of Medicine Atatürk University Erzurum Türkiye; ^4^ Department of Physiotherapy and Rehabilitation, Faculty of Health Sciences University of Yalova Türkiye

**Keywords:** Antioxidant Defense, D‐Carvone, renal damage

## Abstract

Lithium‐induced nephrotoxicity presents a major health concern due to its detrimental impact on renal function, as is frequently observed in clinical and experimental settings. Despite various efforts to address this issue, effective treatment options remain limited. D‐Carvone, a natural compound with antioxidants and anti‐inflammatory properties, has demonstrated promise in reducing oxidative stress and inflammation. The present study aimed to explore the therapeutic impact of D‐Carvone in alleviating lithium‐induced nephrotoxicity, with a particular focus on oxidative stress indicators, apoptosis, inflammation, and histological changes in renal tissue. To this end, a total of 24 male rats were divided into four groups: Control, Lithium, Lithium + D‐Carvone (20 mg/kg daily), and D‐Carvone alone, with six animals per group. Lithium‐induced nephrotoxicity was evaluated over 14 days. Oxidative stress was measured by evaluating malondialdehyde (MDA), glutathione (GSH) levels, and superoxide dismutase (SOD) activity. The expression of Caspase‐3, Bcl‐2, HO‐1, TLR4, NRF2, and NF‐κB‐p65 was analyzed to assess apoptotic, inflammatory, and oxidative stress pathways. A histopathological examination of the kidney cortex and medulla was conducted. The results revealed that lithium administration caused significant histological damage, increased oxidative stress, and altered apoptotic and inflammatory protein expression. D‐Carvone treatment reduced lipid peroxidation, restored apoptotic protein balance, alleviated inflammation, and protected renal tissue. These findings highlight the potential of D‐Carvone as an adjuvant therapy for mitigating the nephrotoxic effects of lithium.

## Graphical abstract

Lithium administration led to significant oxidative stress, histopathological kidney damage, and elevated apoptotic and inflammatory markers in rats. D‐Carvone treatment markedly reduced lipid peroxidation, restored antioxidant enzyme activity, and alleviated apoptosis and inflammation. These findings suggest that D‐Carvone exerts protective effects against lithium‐induced nephrotoxicity and may serve as a promising adjunct therapy.

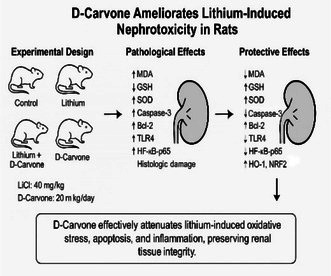

## 1 Introduction

Lithium, a first‐line treatment for bipolar disorder, is recommended for both acute and maintenance phases of therapy. However, as bipolar disorder typically emerges in early life and necessitates extended pharmacological treatment, potential adverse effects and resistance to psychotropic medicines provide considerable challenges (Tohen et al. 2009). Renal toxicity from lithium has a significant negative impact involving complicated mechanisms, including oxidative stress, inflammation, and death. Although the precise mechanisms of lithium‐induced kidney damage remain unclear, prolonged lithium exposure was previously reported to be associated with nephrotoxicity (Duni et al. 2019; Piko et al. 2023; Ranasinghe et al. 2023).

Alterations in antioxidant enzyme activity relate to lithium‐induced kidney damage. Although glutathione (GSH) levels and superoxide dismutase (SOD) activity are lowered, malondialdehyde (MDA) levels increase (Alsawaf et al. 2022). These changes reduce the kidney's ability to control reactive oxygen species (ROS), activating the NF‐κB signaling pathway and producing inflammatory cytokines like TNF‐α and IL‐1β (Liu et al. 2023). Previous clinical studies indicated that a considerable proportion of patients undergoing prolonged lithium therapy develop chronic kidney disease (Bassilios et al. 2008; Bendz et al. 2001; Bocchetta et al. 2013), with some advancing to end‐stage renal failure (Close et al. 2014; Tredget et al. 2010).

In addition to pharmacological strategies, non‐pharmacological interventions were also investigated to prevent lithium‐induced renal damage. For instance, Saberi et al. (2025) showed that endurance exercise at different intensities significantly enhanced glomerular filtration rate (GFR), reduced TNF‐α levels, and increased IL‐10 and SIRT1 expression in a rat model of lithium‐induced nephropathy. The findings further revealed that exercise upregulated renal AQP2 and GSK3β levels, both of which were suppressed by lithium (Saberi et al. 2025). This highlights the potential value of lifestyle‐based interventions, such as a controlled exercise regimen, alongside pharmacologic agents such as D‐Carvone for reducing lithium‐induced renal dysfunction.

Extended lithium exposure was reported to induce renal injury in both animal and human investigations, characterized by renal tubule constriction, interstitial fibrosis, and compromised glomerular filtration (Davis et al. 2018). While the precise processes remain inadequately clarified, oxidative stress undeniably contributes to renal injury (Rashid et al. 2023).

In this context, D‐Carvone, a molecule with antioxidant (Rajeshwari and Raja 2015) and anti‐inflammatory (Zhao and Du 2020) characteristics, demonstrated potential as a therapeutic intervention for lithium‐induced kidney injury. Although managing bipolar disease depends on it, lithium was previously linked to renal impairment (Boivin et al. 2023; Zhang et al. 2022). D‐Carvone has demonstrated potential in reducing harmful impacts by changing important metabolic routes. Superoxide dismutase (SOD) and glutathione (GSH) are two antioxidant enzymes whose activity increases oxidative equilibrium in renal cells (Sadiq et al. 2010). D‐Carvone was also found to reduce carbon tetrachloride‐induced liver fibrosis by lowering oxidative stress and the TGF‐β1/SMAD signaling pathway (Ogaly et al. 2022). A previous study on septic mice found that D‐Carvone improved the lung wet/dry ratio by reducing blood levels of TNF‐α, IL‐1β, and IL‐6, which in turn had anti‐inflammatory advantages (Zhao and Du 2020). Despite conflicting claims, there is a dearth of research on D‐Carvone's ability to mitigate lithium‐induced kidney impairment. Accordingly, the present study seeks to determine whether D‐Carvone can alleviate the inflammation and oxidative stress associated with lithium‐induced kidney damage. By looking at the root causes—namely, oxidative imbalance, inflammation, and death—of kidney damage caused by lithium exposure over an extended period, we might learn whether D‐Carvone would be a solution.

Our goal is to learn how D‐Carvone works molecularly by looking at how levels of Caspase‐3, Bcl‐2, HO‐1, TLR4, NRF2, and NF‐κB‐p65 protein expression in renal tissues change after 14 days of treatment. To better understand the mechanisms causing lithium‐induced kidney injury, we looked at the expression of TLR4 and HO‐1. By controlling these genes, inflammation and oxidative stress can be drastically decreased. However, TLR4 activation enhances inflammatory pathways and exacerbates renal failure (Lucas and Maes 2013), whereas HO‐1 aids cells in defending against oxidative damage (Loboda et al. 2016). Regulating these genes can considerably reduce inflammation and oxidative stress. While HO‐1 protects cells from oxidative damage, TLR4 activation exacerbates inflammatory pathways, worsening renal failure (Li et al. 2021).

In this study, we selected chemicals known for their role in the pathophysiology of kidney injury and their ability to influence oxidative stress and inflammatory processes linked to lithium toxicity. Because of their crucial functions in regulating apoptosis, oxidative stress, and inflammation, all of which play a significant role in the kidney damage caused by lithium, we focused on Caspase‐3, Bcl‐2, NRF2, and NF‐κB‐p65. One protein, Caspase‐3, controls cell death, while another, Bcl‐2, controls cell survival (Hussar 2022). D‐carvone may be able to minimize kidney damage by regulating pathways that reduce inflammation and oxidative stress, boost cell viability, and lower apoptosis, as NF‐κB‐p65 increases inflammation and NRF2 decreases oxidative stress (Gao et al. 2022). D‐Carvone mitigates lithium‐induced kidney damage; how it does this is the subject of future histological investigations. In this study, our goal was to understand the therapeutic efficacy of D‐Carvone in reducing lithium‐induced nephrotoxicity.

## 2 Materials and Methods

### 2.1 Animals

We used 12‐week‐old male adult Sprague Dawley rats from the Atatürk University Experimental Research Center (ATADEM). The rats were housed individually in controlled environments with unrestricted access to regular commercial feed and water. The animals in the lithium chloride (LiCl) treatment groups received a commercially prepared meal enhanced with 40 mmol LiCl per kilogram of dry feed (Erbaş, Gelen, and Öztürk 2024). Arden Research and Experiment Company in Ankara, Türkiye, produced the LiCl‐enriched dinners. We used 98% pure D‐Carvone, obtained from Thermo Fisher Scientific (USA).

### 2.2 Experimental Design and Groups

**FIGURE 1:**
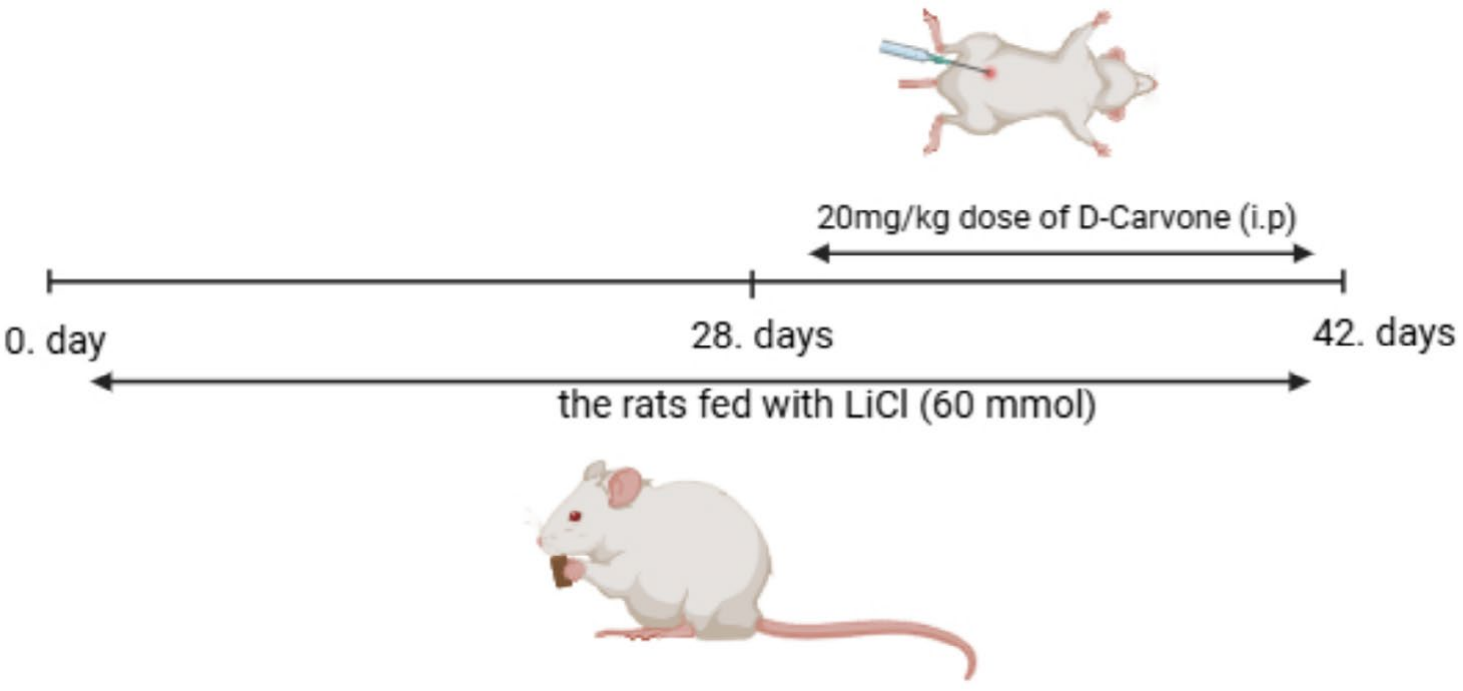
Procedure and treatment methodology for the study.

All experiments were approved by the Atatürk University Animal Experiments Local Ethics Committee under decision number 2024/09–221. A total of 24 adult male rodents from ATADEM were randomly assigned to four groups, six animals per group, according to their body weight. The rodents in the allotted groups consumed a 28‐day lithium chloride (LiCl)‐enriched diet that contained 40 mmol LiCl per kilogram of dry chow during the study (Erbaş, Üstündağ et al. 2024). Subsequently, a 14‐day course of intraperitoneal D‐Carvone treatment at a dosage of 20 mg/kg was initiated (see Figure 1). In scientific research, accurate dosage calculation is crucial to enhance treatment efficacy and reduce the likelihood of harmful effects. Previous research indicated that a dosage of 20 mg/kg of D‐carvone significantly affects many physiological parameters, including oxidative stress and inflammatory markers, more than the 5 and 10 mg/kg doses (Raja 2015). Considering the low levels of oxidative damage and inflammatory mediators, the dosage is a crucial determinant of medicinal efficacy. Investigating the possible benefits of D‐carvone inside a therapeutic range fit for its use is considerably easier because of the constant and trustworthy results this dosage generates. This dosage selection ensures that D‐Carvone's potential benefits are evaluated within an optimal therapeutic range, enabling reliable and consistent results. Sevoflurane was used to anesthetize all animals after the 14‐day treatment period. Upon complete anesthetization, the animals' kidney tissues were removed. While kidney tissue sections were fixed in a 10% buffered formaldehyde solution for histological evaluation, the remaining tissues were cryopreserved at −80°C for further Western blot and biochemical studies. The four different experimental groups, the rats were as follows:

Control Group: This cohort did not receive any treatment.

The D‐Carvone Group: Rats received an intraperitoneal injection of 20 mg/kg D‐Carvone (without lithium), as outlined by (Erbaş, Gelen and Öztürk 2024) to evaluate its effects on renal function and histopathology.

The Lithium Group (LIT): Rats received a diet supplemented with lithium chloride (LiCl) at a concentration of 40 mmol LiCl per kilogram of dry chow (Erbaş, Gelen and Öztürk 2024) for 42 days to test its effects on renal function and histology.

The Lithium + D‐Carvone Group (LIT + D‐Carvone): Rats received concurrent treatment with lithium (40 mmol) and D‐Carvone (20 mg/kg) to examine the possible therapeutic effects of D‐Carvone on lithium‐induced renal injury.

### 2.3 Histopathological Analysis

To ensure the best preservation, kidney tissue from each group of rodents was put in 10% neutral buffered formalin and left there for 72 h. The tissues were treated with graded alcohol, then the alcohol was removed using xylene, and after fixing, they were embedded in paraffin. Five μm‐thick sections were obtained from paraffin blocks using a Leica RM2125 RTS microtome for histological examination. The sections were stained with Crossman's modified Mallory's Trichrome to yield a detailed representation of tissue injury. Histopathological abnormalities were rated on a scale of 0 to 4, where 0 indicated no damage, 1 = mild damage, 2 = moderate damage, 3 = severe damage, and 4 = extensive damage (Erbaş, Üstündağ et al. 2024; Kara et al. 2023). A camera‐integrated light microscope (Nikon Eclipse i50, Tokyo, Japan) was used for microscopic examinations.

### 2.4 Biochemical Analysis

We measured glutathione (GSH), malondialdehyde (MDA), and superoxide dismutase (SOD) activity in renal tissue using enzyme‐linked immunosorbent assays (ELISAs). The tissues were lysed for 5 min at 30 Hz using a Qiagen Tissue Lyser II after cryo treatment with liquid nitrogen. The samples were combined with PBS (pH 7.4) and subsequently homogenized for 20 s at 30 Hz. Centrifugation at 3000 rpm produced supernatant. Malondialdehyde (MDA), glutathione peroxidase (GSH), superoxide dismutase (SOD), and tissue protein were evaluated using enzyme‐linked immunosorbent assay (ELISA) kits from YLbiont. The manufacturer's standards were adhered to in all investigations. MDA, GSH, and SOD were detected at 450 nm, while tissue protein was detected at 595 nm using a spectrophotometer (BioTek, vQuant, USA). In addition, serum lithium concentrations were measured using the Abcam Lithium Assay Kit (Colorimetric, ab235613) according to the manufacturer's instructions to verify lithium exposure and to support the evaluation of lithium‐induced renal damage.

### 2.5 Western Blot Analysis

The kidney tissue samples were preserved at −80°C until they were prepared for Western blot analysis. Frozen tissue specimens were initially ground in liquid nitrogen for optimal preservation, then homogenized in a radioimmunoprecipitation assay (RIPA) buffer supplemented with protease and phosphatase inhibitors to assess the relative protein levels of Caspase‐3, Bcl‐2, HO‐1, TLR4, NRF2, and NF‐κB‐P65. A tissue lyser device (Qiagen) operating at 30 Hz for 20 s aided in this procedure. After extracting the proteins from kidney tissues, the concentration of proteins was determined using the Pierce BCA protein assay kit (Thermo Scientific). Next, 30 μg of protein from each sample was resolved using a 10% SDS‐PAGE gel and subsequently transferred onto a PVDF membrane. The membranes were subsequently treated with 5% bovine serum albumin for 90 min at ambient temperature to inhibit non‐specific binding. After the blocking process, the membranes were treated overnight at 4°C with primary antibodies specific to the target proteins (see Table 1). Following incubation with primary antibodies, the membranes were washed with Tris‐buffered saline containing Tween (TBST) to eliminate any unbound antibodies. The samples were then treated with horseradish peroxidase‐conjugated secondary antibodies (Santa Cruz, sc‐2004/sc‐2005) for 90 min at room temperature. Protein bands were seen using an enhanced chemiluminescence reagent (Thermo, 3405) and documented with Image Lab Software (Bio‐Rad, Hercules) for subsequent quantitative assessment.

**TABLE 1:** Antibodies used in Western blot analysis.

Antibody	Catalog no	Western dilution
Caspase‐3	DF9222, Affinity Biotech	1/1000
Bcl‐2	DF9225, Affinity Biotech	1/1000
HO‐1	AF5222, Affinity Biotech	1/1000
TLR4	AF5231, Affinity Biotech	1/1000
NRF2	AF5164, Affinity Biotech	1/1000
NF‐κB‐P65	AF5006, Affinity Biotech	1/1000
Beta Actin	sc‐47,778, Santa Cruz	1/2000
Goat anti‐rabbit	sc‐2004/sc‐2005, Santa Cruz	1/2000

### 2.6 Statistical Analysis

Upon completion of the study, the data were analyzed using the SPSS 22.00 statistical program. The results were expressed as mean ± standard deviation (SD). Parametric One‐Way ANOVA was run to evaluate the normality of the data distribution, followed by Tukey's test. A *p*‐value below 0.05 was statistically significant.

## 3 Results

### 3.1 Histopathological Changes

The results showed that both the control and D‐Carvone groups exhibited normal histological structures in their kidneys. The glomeruli and tubules in the cortical and medullary regions appeared normal. The lithium (LIT) group exhibited notable glomerular and tubular architectural abnormalities.

These changes included glomerular atrophy, cell architecture changes in the cortical and medullary tubules, tubular integrity problems, and some swelling in the cortex. The D‐Carvone group had a mean score of 0.5833 ± 0.2787, and the kidneys looked almost normal, identical to those in the control group (mean score of 0.6167 ± 0.3251).

The mean score in the LIT group significantly increased to 3.772 ± 0.1078 (*p* < 0.001). This finding suggests that lithium causes significant harm to kidney tissues. The LIT + D‐Carvone group demonstrated a mean score of 3.117 ± 0.2317, which was lower than that of the LIT group; however, this difference was not statistically significant (*p* > 0.05).

This suggests that D‐Carvone partially mitigates the damage caused by lithium but does not result in complete recovery. The histopathological examination showed changes in the structure of the kidneys, including glomerular shrinkage, tubular degeneration, and edema. These findings suggest that the changes reduce the kidney's ability to control reactive oxygen species (ROS), activating the NF‐κB signaling pathway and producing inflammatory cytokines like TNF‐α and IL‐1β. Kidney tissue morphology was very different.

**FIGURE 2:**
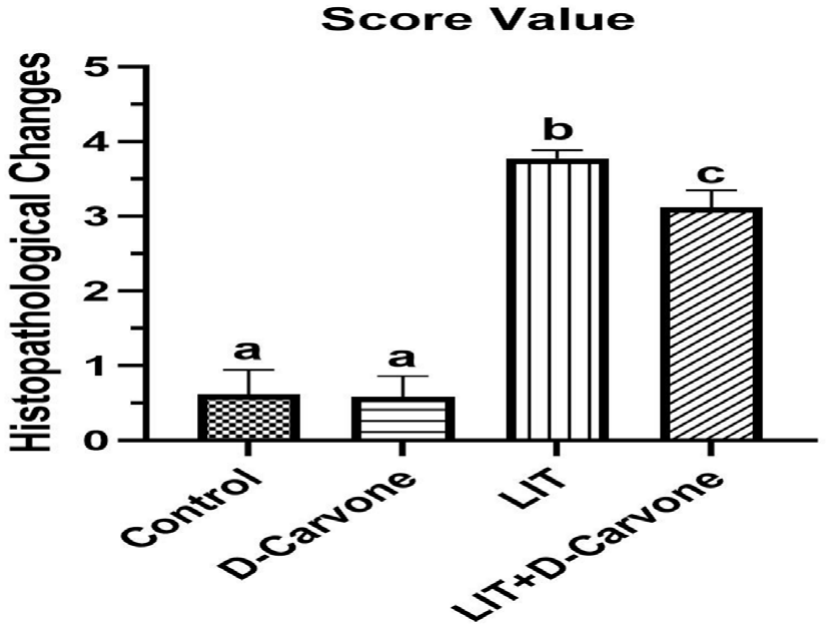
Histopathological evaluation of renal tissue across all groups. Score values: (0) No discernible injury; (1) negligible damage; (2) moderate damage; (3) significant damage; (4) severe damage. Values are expressed as mean ± SD (*n* = 6) and examined via one‐way ANOVA followed by Tukey's test. The disparity between the groups is statistically significant, as indicated by b and c for *p* < 0.05, whereas “a” signifies a non‐significant difference.

**FIGURE 3:**
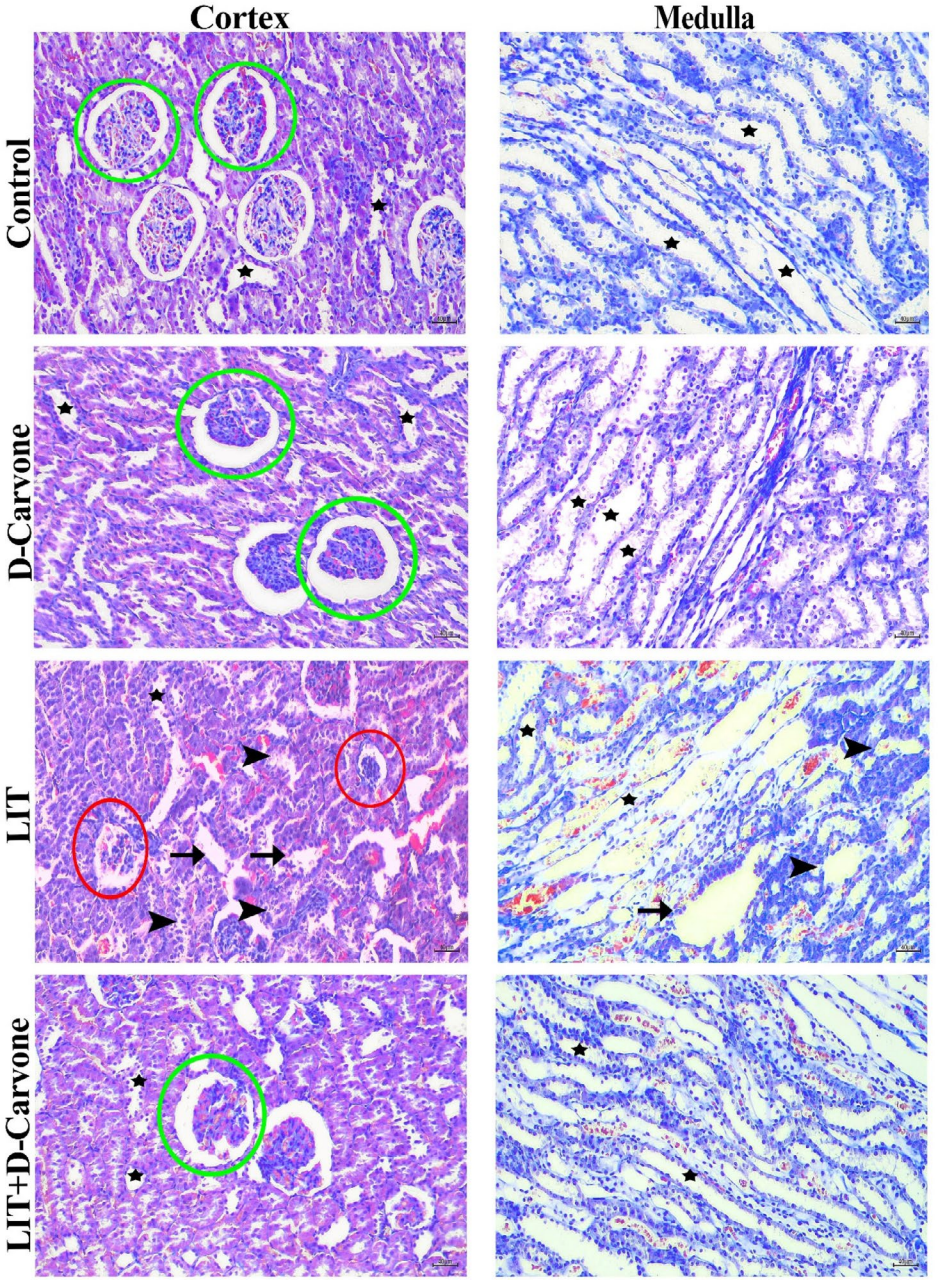
Comprehensive histopathological evaluations for all experimental groups. Green cycle: Glomerulus; black stars: Kidney tubules; black arrowhead: Degenerative tubules; black arrow: Edema; red cycle: Degenerative glomerulus. Crossman modified Mallory's triple staining, magnification X200.

Kidney tissues in the experimental groups treated with Lithium and D‐Carvone showed markedly improved morphological preservation. While tubular degeneration decreased, the glomerular structure remained largely intact. These findings suggest that D‐Carvone can protect against lithium‐induced nephrotoxicity. Thorough histological evaluations of each experimental group corroborate the protective effects (see Figures 2, 3).

### 3.2 Comparative Evaluation of Serum Lithium Levels

**FIGURE 4:**
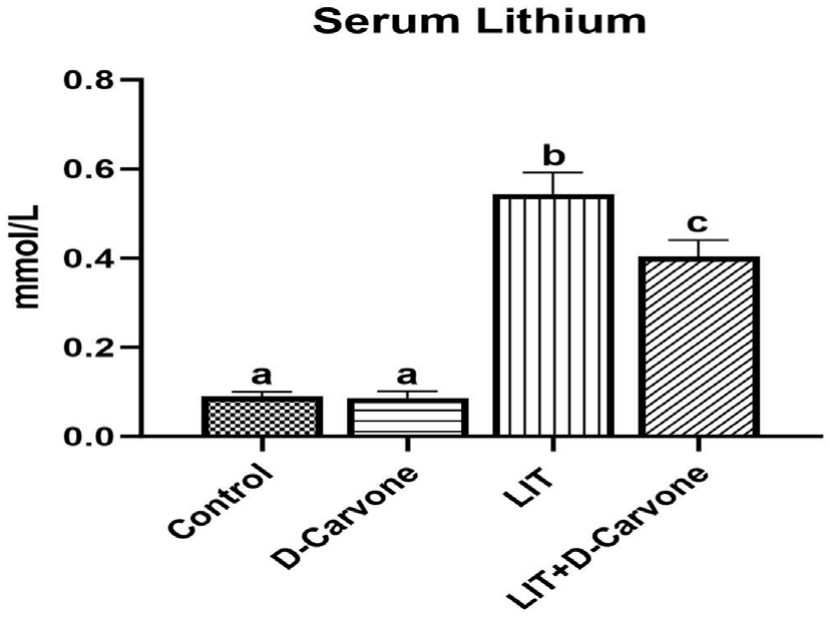
The distribution of serum lithium levels was examined across the groups. Statistically significant differences were observed between groups marked with different letters. (a) No significant difference was found between the control and D‐carvone groups (*p* > 0.05); (b) a significant increase was observed in the LIT group (*p* < 0.0001); (c) significant increase was observed in the LIT + D‐Carvone group (*p* < 0.0001).

Statistical comparisons demonstrated that there was no significant difference between the Control and D‐Carvone groups regarding serum lithium levels (both denoted with the letter “a,” *p* > 0.05), indicating that D‐Carvone alone did not affect systemic lithium concentrations. By contrast, the LIT group exhibited the highest serum lithium levels among all groups. It showed a statistically significant difference from the others (denoted with the letter “b,” *p* < 0.05), confirming that systemic lithium administration leads to a marked elevation in serum levels. Importantly, co‐administration of D‐Carvone with lithium (LIT + D‐Carvone group) resulted in significantly lower serum lithium levels as compared to those in the LIT group alone (denoted with the letter “c,” *p* < 0.05). This finding suggests that D‐Carvone may reduce lithium bioavailability or affect its metabolism and elimination. The results of our analysis of serum lithium levels revealed statistically significant differences among the experimental groups (*p* < 0.05), with the mean ± standard deviation values provided in Table 2 and Serum lithium levels for all groups presented in Figure 4.

**TABLE 2:** The mean and standard deviation values of serum lithium measurements for all groups.

Group	Mean ± Standard Deviation
Control	0.09000 ± 0.01000
D‐Carvone	0.08667 ± 0.01528
LIT	0.5440 ± 0.04827
LIT+D‐Carvone	0.4040 ± 0.03647

*Note:* Serum lithium levels were presented as mean ± standard deviation for each group. A statistically significant difference was observed when compared to the Control group (a), the D‐Carvone group (b), the LIT + D‐Carvone group (c), and the LIT group (d), respectively.

### 3.3 Oxidative Stress Results

**FIGURE 5:**
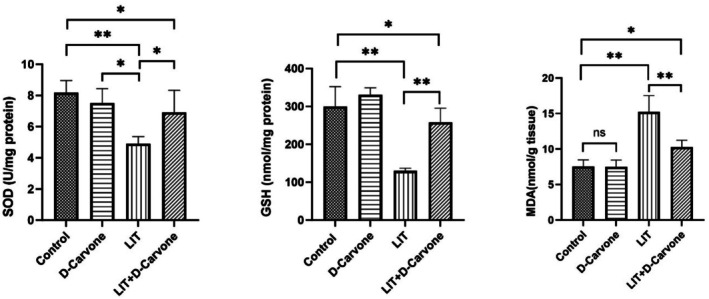
Comprehensive evaluation of oxidative stress indicators across all experimental groups, encompassing MDA (malondialdehyde), GSH (glutathione), and SOD (superoxide dismutase). Presented as mean ± SD (*n* = 6), values are subjected to one‐way ANOVA then Tukey's test. For *p* < 0.05, the groups show a statistically significant difference represented by b and c; “a” indicates a non‐significant difference.

During the tissue oxidative parameter analysis, MDA levels were significantly elevated in the lithium‐treated groups as compared to those in the control and D‐Carvone‐treated groups (*p* < 0.05). This augmentation signifies elevated oxidative stress linked to lithium intake. The groups administered both lithium and D‐Carvone demonstrated decreased MDA levels, indicating that D‐Carvone may alleviate lipid peroxidation caused by lithium treatment. The activity of SOD, essential for neutralizing superoxide radicals, decreased in the lithium‐treated groups, supporting the concept of lithium‐induced oxidative stress. In the LIT + D‐Carvone groups, SOD activity was maintained more effectively, suggesting the potential antioxidative role of D‐Carvone. Glutathione (GSH) levels, which reflect antioxidant capacity, were reduced in the lithium group, suggesting a decline in antioxidant defenses (*p* < 0.05). However, the LIT + D‐Carvone cohort demonstrated increased GSH levels as compared to lithium monotherapy, indicating a possible therapeutic effect of D‐Carvone on antioxidant status. Figure 5 presents a detailed analysis of MDA, GSH levels, and SOD activity.

### 3.4 Western Blot Analysis Results

As compared to the control group, in the lithium‐treated (LIT) group, the Western blot analysis of kidney tissue showed a notable rise in NF‐κB‐P65 expression (*p* < 0.05). The D‐Carvone and LIT+D‐Carvone groups clearly showed a decrease in this augmentation. NRF2 protein expression was substantially reduced in the LIT group as compared to the control group (*p* < 0.05). After D‐Carvone treatment, these levels increased and exceeded those observed in the LIT group in approaching control values. This suggests that D‐Carvone may possess properties that mitigate oxidative damage. Expression of TLR4 protein was considerably higher in the LIT group than in the control group (*p* < 0.05).

**FIGURE 6:**
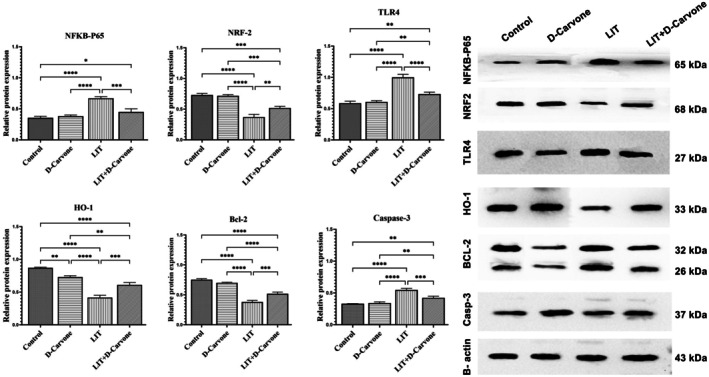
Protein expression levels of Caspase‐3, Bcl‐2, HO‐1, TLR4, NRF2, and NF‐κB‐P65 were assessed. The values are expressed as mean ± standard deviation (*n* = 6). The letters denote the statistical disparities among the groups. The results were subjected to one‐way ANOVA, subsequently followed by Tukey's test. The groups demonstrate a statistically significant difference, as indicated by b and c for *p* < 0.05, while “a” signifies a non‐significant difference.

Furthermore, the D‐Carvone and LIT+D‐Carvone groups exhibited significantly reduced levels, indicating that D‐Carvone may attenuate the inflammatory response. While the D‐Carvone and LIT+D‐Carvone groups exhibited reduced levels, the LIT group (*p* < 0.05) demonstrated a significant increase in HO‐1 expression (*p* < 0.05). Bcl‐2 expression, associated with cell survival, was significantly reduced in the LIT group (*p* < 0.05); however, D‐Carvone therapy mitigated this decrease. Bcl‐2 levels in the LIT+D‐Carvone group were higher compared to those in the LIT group, suggesting that D‐Carvone may improve cellular viability. Furthermore, while Caspase‐3 expression significantly increased in the LIT group (*p* < 0.05), these levels decreased in both the D‐Carvone and LIT + D‐Carvone groups, approaching control values. D‐Carvone may inhibit apoptosis. Figure 6 presents protein bands, expression levels, and the results of comparative analyses.

## 4 Discussion

The results of the present study demonstrated the protective effects of D‐carvone against lithium‐induced kidney damage, showing that D‐carvone alleviates oxidative stress and inflammation, thereby contributing to the preservation of kidney tissue. Lithium treatment increased oxidative stress, leading to pathological changes in the kidneys, while D‐carvone significantly mitigated these harmful effects. The results of our histological analysis revealed that D‐carvone alleviated structural damage, such as glomerular shrinkage, tubular abnormalities, and edema induced by lithium treatment, resulting in the preservation of kidney function.

Lithium treatment is known to induce oxidative stress by disrupting the balance between reactive oxygen species (ROS) and antioxidant defenses, contributing to kidney damage (Nocella et al. 2019). Lithium treatment was previously reported to increase malondialdehyde (MDA) levels, reduce superoxide dismutase (SOD) activity, and decrease glutathione (GSH) levels (Ommati et al. 2021; Toplan et al. 2016). These changes result in oxidative damage to kidney cells, leading to impaired kidney function (Ommati et al. 2021). However, D‐carvone treatment significantly improved these oxidative stress parameters. D‐carvone increased GSH levels, reduced MDA levels, and thus decreased lipid peroxidation while also enhancing SOD activity. Taken together, these findings underscore D‐carvone's potent antioxidant properties and its ability to mitigate oxidative damage in the kidneys.

D‐carvone's impact on oxidative stress was extensively documented in the literature (Rajeshwari and Raja 2015); (Erbaş, Gelen and Öztürk 2024). D‐carvone enhances the expression of key antioxidant response proteins, including Nrf2 and HO‐1. Nrf2 is a transcription factor that increases the efficiency of cellular antioxidant defenses (Ngo and Duennwald 2022). D‐carvone was found to increase Nrf2 levels in the kidneys, thereby strengthening cellular defense and reducing structural damage to the kidneys.

Lithium treatment also exacerbates kidney inflammation, characterized by excessive expression of TLR4 proteins and NF‐κB‐p65 (Fu et al. 2024). Specifically, D‐carvone treatment was found to reduce TLR4 and NF‐κB‐p65 expression, thereby modulating the inflammatory response. HO‐1 is an enzyme that plays a crucial role in reducing oxidative damage and regulating cellular stress (Yachie 2021). D‐carvone increased HO‐1 levels, thereby strengthening cellular defenses and protecting kidney cells from oxidative damage.

Another mechanism by which D‐carvone prevents lithium‐induced kidney cell death is by reducing the expression of Caspase‐3, a key protein involved in inducing cell death whose overexpression leads to increased cellular apoptosis. D‐carvone reduced Caspase‐3 expression and increased Bcl‐2 protein levels, thereby decreasing cell death and preserving cellular integrity (Mohamed and Younis 2022); Ogaly et al. 2022; (Erbaş, Gelen and Öztürk 2024). Taken together, these findings support the anti‐apoptotic properties of D‐carvone, suggesting its role in mitigating renal injury through modulation of oxidative stress, inflammation, and apoptotic pathways.

In line with our findings, previous studies revealed that lithium exposure increases oxidative stress and triggers inflammation, resulting in significant renal damage (Rashid et al. 2023).

Similarly, Saberi et al. (2025) demonstrated that various intensities of endurance exercise significantly improved renal function in lithium‐treated rats by reducing TNF‐α levels, enhancing IL‐10 and SIRT1 expression, and upregulating AQP2 and GSK3β proteins. These results support the notion that non‐pharmacological interventions, such as exercise, may exert protective effects through mechanisms similar to those of D‐Carvone, particularly by modulating inflammation and oxidative pathways.

Taken together, both pharmacological and behavioral strategies may offer complementary approaches in managing lithium‐induced nephrotoxicity.

Finally, we also observed that serum lithium levels were significantly different between the experimental groups (*p* < 0.05). There was no significant difference in serum lithium levels between the control and D‐carvone groups, whereas the lithium (LIT) group showed significantly higher serum lithium levels than the other groups. This finding corroborates that lithium treatment increases serum lithium levels, thereby contributing to its toxic effects. The toxic effects of lithium on the kidneys are associated with increased oxidative stress, and elevated serum lithium levels can exacerbate renal dysfunction (Ossani et al. 2019). However, serum lithium levels were significantly reduced in the D‐carvone and lithium + D‐carvone (LIT + D‐Carvone) groups. This finding suggests that D‐carvone may modulate lithium bioavailability or enhance its elimination, thus contributing to the reduction of kidney damage. Taken together, our results suggest that D‐carvone helps to protect the kidneys by lowering serum lithium levels and mitigating lithium‐induced toxicity (Gitlin and Bauer 2023; Rej et al. 2013).

### 4.1 Limitations and Future Directions

This study has several limitations. First, pharmacokinetic studies examining the impact of D‐carvone on lithium bioavailability have not yet been conducted. Accordingly, further research is needed to elucidate the mechanisms underlying the changes in serum lithium levels. Second, the long‐term effects of D‐carvone in clinical settings have not yet been evaluated, so its efficacy in humans should be determined. Further studies should also explore the therapeutic potential of D‐carvone in larger cohorts and conduct a more comprehensive analysis of its pharmacological profile.

## Author Contributions

**Semin Gedikli:** conceptualization (equal), data curation (equal), investigation (equal), methodology (equal). **Deniz Tekiner:** conceptualization (equal), data curation (equal), formal analysis (equal), investigation (equal), methodology (equal). **Hülya Kara:** conceptualization (equal), data curation (equal), investigation (equal), methodology (equal). **Saime Özbek Şebin:** data curation (equal), formal analysis (equal), investigation (equal). **Tubanur Aslan Engin:** data curation (equal), investigation (equal).

## Ethics Statement

The experimental protocols fully complied with the ethical standards of the Atatürk University Local Ethics Committee for Animal Experiments (Decision no: 2024/09–221).

## Conflicts of Interest

The authors declare no conflicts of interest.

## Data Availability

Data will be made available on request.
